# Recent Advances Towards Point-Of-Care Applications of Surface-Enhanced Raman Scattering Sensing

**DOI:** 10.3389/fchem.2021.714113

**Published:** 2021-08-09

**Authors:** Steven Quarin, Pietro Strobbia

**Affiliations:** Department of Chemistry, University of Cincinnati, Cincinnati, OH, United States

**Keywords:** point-of-care (POC), surface-enhanced Raman scattering (SERS), microRNA (miRNA), plasmonics, diagnostics

## Abstract

The ability to accurately diagnose at the point of care is crucial in many pathologies. However, current standard diagnostic practices can only be performed in specialized health or laboratory settings. To move diagnostic methods from a specialized lab to the point of care many alternate methods have been developed and proposed. Among them surface-enhanced Raman scattering (SERS) sensing offers advantageous features, such as simultaneous detection of multiple biotargets and increased accuracy. Many groups have been working towards the translation of SERS sensing methods from the lab to the point of need. In this mini review, we discuss interesting and recent developments in this effort, focusing on how different sensing mechanism can be used in point-of-care testing applications of SERS.

## Introduction

The ability for accurately point-of-care (POC) diagnosis is crucial for many diseases to ensure early intervention and/or reduce the disease spread. However, many of the current standard diagnostic practices can only be performed in large health centers and/or have long turnaround times (Li and Wu, 2013; Vahrmeijer et al., 2013). Common examples are the detection of cancers, performed with MRI and CT scans or via histopathological review. MRI or CT require the expensive instrumentation and are only available in large hospitals, while histopathological review requires multiple days and expert pathologists (Demétrio de Souza França et al., 2020). For the case of infectious pathogens, the current standard diagnostic practice is not suitable for point-of-care testing (POCT). Such diseases are commonly detected with biological or biomolecular diagnostic methods such as culturing cells or polymerase chain reaction (PCR) to detect the specific genetic signature of the pathogen (Influenza Signs and Sympt, 2021; Malaria Diagnosis (United States), 2019). Cell cultures are very useful for bacterial infections diagnosis as they can give additional information such as antibiotic resistance; however, this method requires multiple days and specialized laboratory settings. Similarly, PCR also requires specialized settings, personnel and instrumentation.

To move diagnostic methods from a specialized lab to the POC, alternate methods have been proposed. Among them, methods based on optical phenomena are ideally suited to work at the POC, due to the possibility for non-contact measurements and the use of the ubiquitous silicon chips as a detector. The most used optical techniques in POCT are colorimetry and fluorimetry (Zhu et al., 2013). Both these methods are based on optical phenomena with broad spectral responses, with bandwidths on the order of 100 nm. This feature limits the number of simultaneously observable peaks to two or three, thereby limiting multiplexed detection of biotargets. In addition, broad spectral profiles hinder the extraction of the sensor optical response from background interferences. Example of interferences are autofluorescence or external light and contaminants, reducing the achievable accuracy especially in POC settings. Conversely, Raman scattering is an optical phenomenon characterized by very sharp spectral features, given by its vibrational nature. The sharp Raman peaks permit to detect numerous analytes simultaneously and to clearly distinguish background from Raman signal (Faulds et al., 2008; Strobbia et al., 2021). However, a critical issue with Raman is the inherently low signal that significantly reduces the sensitivity for analyte detection.

While the sensitivity of spontaneous Raman does not permit its use in POCT diagnostic applications, surface-enhanced Raman scattering (SERS) can be used to exploit this vibrational spectroscopy in these settings. SERS is a plasmonic phenomenon due to the amplification of scattering on the surface of metallic nanoparticles. The amplification arises from the localized surface plasmon resonance, a collective oscillation of conductive band electrons produced by the interaction with light at a specific wavelength (Moskovits, 2005). This enhancement or amplification of the Raman signal permits to take advantage of the feature of Raman spectroscopy (e.g., multiplexing, accuracy) for diagnostic purposes.

SERS can be used in diagnostic methods in multiple ways divided between intrinsic and extrinsic SERS sensing. In intrinsic SERS sensing, samples are mixed or deposited directly on a SERS active surface and the SERS spectrum from its components can be detected. The analysis of these data usually requires advanced statistical methods, such as principal component analysis (PCA) or other machine learning techniques. Recent interesting examples have shown the possibility of detecting cancer-related exosomes, circulating tumoral DNA and specific bacterial infections (Feng et al., 2010; Mühlig et al., 2016; Shin et al., 2018). These methods are easy to implement and usually requires very little sample preparation. However, the complexity of the signal makes it almost impossible to decipher which components of the sample are generating the diagnostic answer, making it a harder sell to health professionals and regulatory boards. Alternatively, SERS nanoparticles can be used as tags (SERS-tags) in molecular assays in extrinsic SERS sensing. SERS-tags are nanoparticles coated with a Raman reporter molecule with known signal. These methods can use mechanisms equivalent to current standard practices, such as lateral flow, ELISA or PCR assays, but with a transduction based on SERS. This feature makes them more likely to be adopted clinically and the current process of translating these methods from the lab to POCT is currently ongoing.

This mini review will discuss recent developments in SERS for POCT diagnostic applications, focusing on recent advances in extrinsic SERS sensing. This review is not meant to cover the full landscape of the field of POCT but only recent and interesting developments that impactfully advance the translation of SERS to POC diagnostic applications.

## Discussion

### Advances in Aggregation-Based Surface-Enhanced Raman Scattering Assays

The most common sensing mechanism used for SERS is the aggregation of SERS-tags induced by a target biomolecule. Receptors in this mechanism can be made of DNA, sugar or proteins, detecting a wide range of biomolecules (Graham et al., 2008; Luo et al., 2021). The advantages of using aggregation sensing mechanism are its simplicity combined with a large sensor response. The latter is due to the drastically increased SERS enhancement observed in aggregate compared to single particles, approximately 5 orders of magnitude.

A recent push in molecular diagnostics is the detection of biomarkers in liquid biopsies (i.e., blood, saliva), that can offer a mean for early non-invasive detection of diseases (Larrea et al., 2016). An emerging class of biomarkers are circulating microRNA (miRNA), whose blood or saliva concentration have been associated with specific diagnosis. Usually, a panel of multiple miRNA is needed to have an accurate diagnostic answer. This feature makes SERS ideal for miRNA diagnostics, as SERS can be used for highly multiplexed target detection.

A recent report showed the SERS detection of miRNA-17, which has been noted as biomarker for preeclampsia (Schechinger et al., 2019). This pathology has no preventive testing and is currently only diagnosed when symptoms appear. For the SERS detection, two sets of silver nanoparticles are functionalized with one-half of the complementary DNA strand to the target miRNA. In the presence of the target, the two halves hybridize with the target, causing the nanoparticles to become bound together. This report demonstrated the detection at clinically relevant concentrations (i.e., 1 nM to 1 pM). Furthermore, the detection was demonstrated to work in complex media, which is key to transfer SERS technologies to the POC. Ideally, an assay of this type could be performed by simply adding the sensing SERS-tags directly into a blood sample or after minimal treatment, making it suitable for POC settings.

**FIGURE 1:**
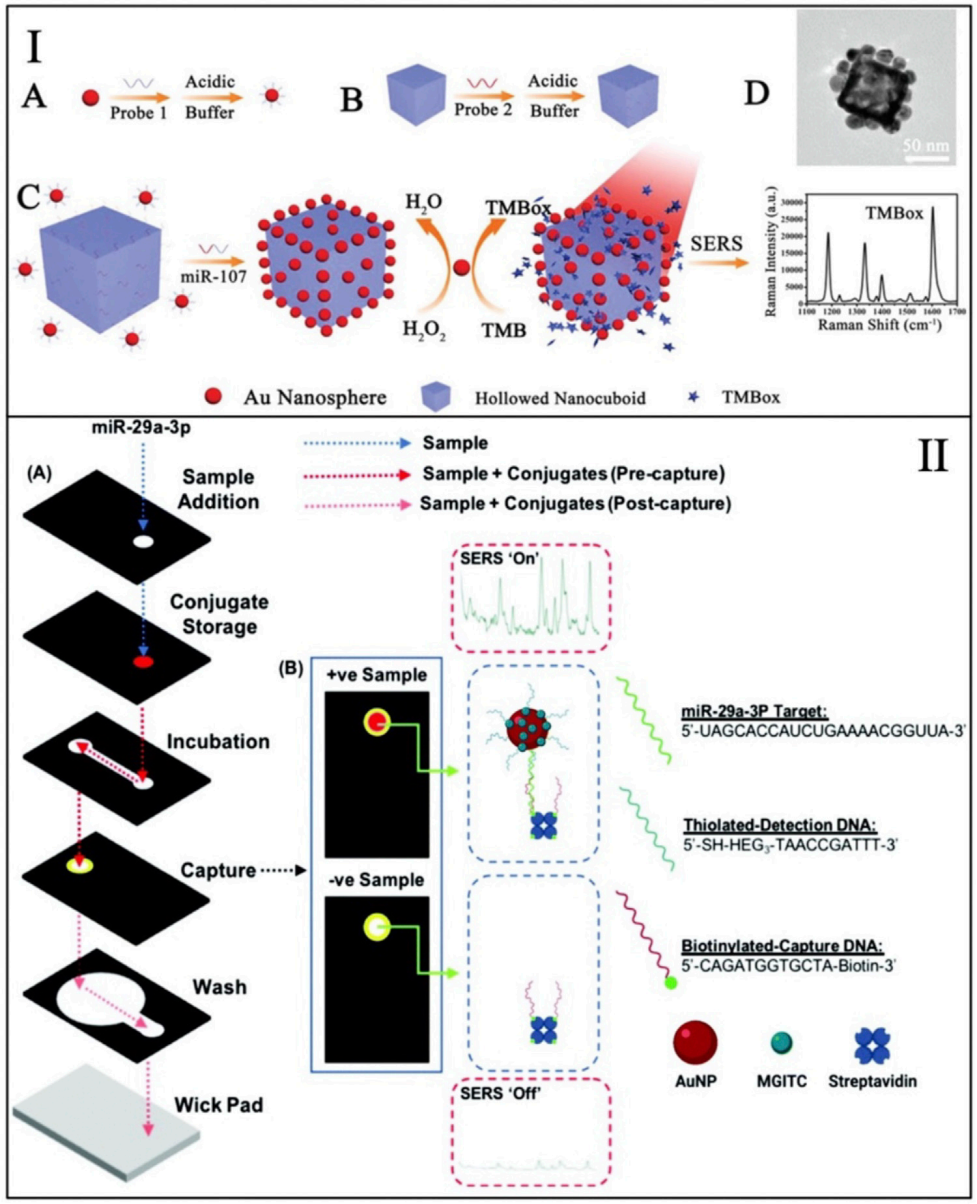
**(I)** Schematic representation of the background-free nanozyme sensing mechanism for the detection of miRNA--107. Adapted from Li et al. (2019). **(II)** Schematics of the 3D-paper fluidic device used for the detection of miRNA-29. Adapted from Mabbott et al. (2020).

A common issue with aggregation-based sensing mechanism is background signal (false positive) from the nonspecific aggregation of nanoparticles. This issue was minimized with a recently developed nanozyme method, where the reporter molecule was catalytically generated (Li et al., 2019). This developed sensing mechanism combines nanospheres and nanocuboids. As described above, both nanoparticles are functionalized with half-complementary sequences and bind in the presence of the target miRNA strand (i.e., miRNA-107). The gold nanospheres act as nanozymes, mimicking peroxidases, and oxidize 3,3′,5,5′-tetramethylbenzidine (TMB) to its oxidized form (TMBox). TMBox generates a stronger SERS signal that is measured as the response from this sensor and can only be detected when the oxidation is coupled with assembly. Figure 1I shows schematics for this sensing mechanism. This strategy permits to have minimal TMBox signal in the absence of target-induced aggregation, therefore boosting the achievable limit of detection. This method demonstrated the detection of miRNA at the femtomolar level and showed the detection of miRNA in clinical samples without the use of target amplification techniques (e.g., PCR).

### Advances in Paper-Based Surface-Enhanced Raman Scattering Assays

A recent interesting advancement towards POCT applications of SERS sensing has been the integration of SERS sensing on paper substrates. Paper substrates have widespread use in rapid diagnostic tests (e.g., lateral flow assays (LFA) such as common pregnancy tests) and are ideally suited for POCT applications due to their cost, storage and simplicity. In these tests, laboratory operations, such as washing and mixing steps, are substituted by the movement of the sample through different functionalized regions of the paper driven by capillary force. Usually, these tests that are based on colorimetry and are characterized by low sensitivity levels, which produces very low diagnostic accuracy for pre-symptomatic detection of diseases. SERS-tags can be integrated in this type of devices, improving the assays by multiplex detection and increased sensitivity. Although the addition of SERS requires a Raman detector, the increased device complexity is well justified by the increase in diagnostic capabilities. Furthermore, many portable Raman detectors have reached the market in recent years for an increasingly reduced cost.

Recent work has shown the detection of miRNA-29 in a paper-based SERS assay. miRNA-29 is a biomarker for cardiovascular diseases and thus is an important target for preventive diagnostics (Mabbott et al., 2020). In this test, both SERS and colorimetric testing are combined for a dual readout testing device. This paper device is a 3D microfluidic paper device that enable multi-step assays that are not easily accessible in LFA. This device was combined with a handheld Raman detector for the SERS analysis. In the presence of miRNA-29, SERS-tags are captured on the surface of paper coated with biotin and functionalized with half-complementary DNA. The captured SERS-tags are coated with a Raman reporter molecule (i.e., MGITC) that gives a specific SERS signal. This mechanism is shown in Figure 1II. The nanoparticles (SERS-tags) change the color on the paper. This change allows the device to be initially read by eye prior to the detection via SERS to get more specific results. The dual reading of this device permitted to directly compare SERS and colorimetry on paper, showing that the SERS results were reproducible and quantifiable in contrast with the colorimetric results read by the device operator.

A SERS-based LFA platform has been recently used for the simultaneous detection of two different DNA targets. In this work, target DNA associated with Kaposi’s sarcoma-associated herpesvirus (KSHV) and bacillary angiomatosis (BA) is detected on a simple LFA design but with SERS-tags used as indicators (Wang et al., 2017). Detection limits below 100 fM were reached for both the biotargets, which is a big boost in sensitivity (5 orders of magnitude) compared colorimetric assays. This demonstration is important as it shows both the increased sensitivity and the possibility for multiplexed detection offered by SERS, proving the rationale for the use of SERS in early disease diagnosis in POCT.

### Advances in Magnetic-Capture Surface-Enhanced Raman Scattering Assays

Magnetic capture is a sensing mechanism that is used in many clinical molecular assays. The advantage of this technique is that the capturing substrate can be controlled and manipulated using a magnet, unlike with microfluidics and LFA where only the flow can be controlled. The increased manipulation permits for more complex series of steps and to integrate the SERS sensing mechanism in autonomous devices. This automation was demonstrated in recent works (Gao et al., 2016; Ngo et al., 2018). A magnetic-driven device was developed to detect malaria RNA directly in blood lysate without sample preparation steps. This method uses nanorattles as SERS-tags, made with nanoparticles composed of a nanosphere casted in a silver cage (Ngo et al., 2018). The assay steps are automated in a capillary tube filled with different solutions. The nanorattles are bound to magnetic nanoparticles in the presence of the target and this complex is washed from unspecifically bound nanorattles through the capillary. The magnetic pellet is then moved to the laser focus to collect the target-dependent SERS signal. This method was able to detect target RNA down to 200-fM concentration and the device was shown to work directly on infected red blood cells, without any purification steps required. The latter result is key for POCT applications of SERS because it shows that such devices could be used directly with blood. Similar autonomous magnetic devices have been developed for the detection of prostate-specific antigen in a microfluidic system, showing capabilities for different biotargets (Gao et al., 2016).

**FIGURE 2:**
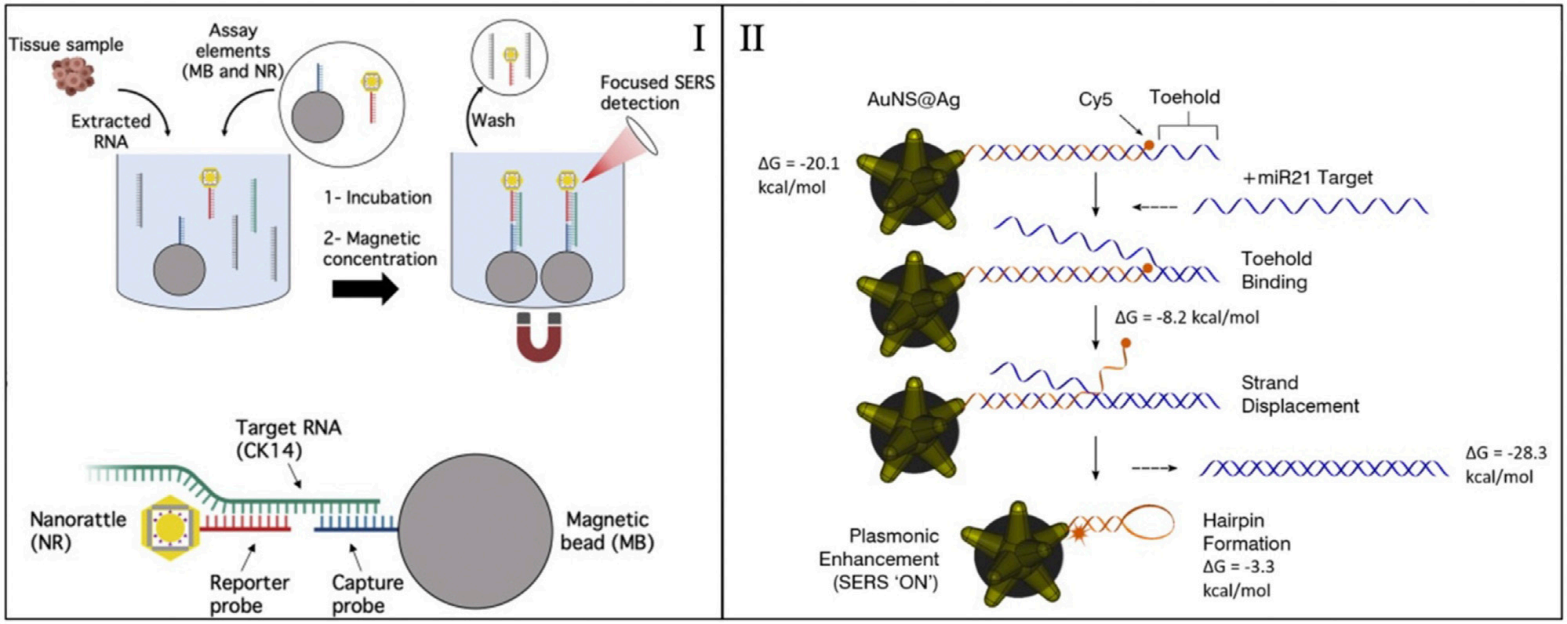
**(I)** Sensing mechanism the magnetic capture of nanorattles for the detection of CK14 RNA. Adapted from Dukes et al. (2020). **(II)** Sensing mechanism of an inverse molecular sentinel. Adapted from Crawford et al. (2020).

A similar sensing mechanism was also applied to detect miRNA biomarkers associated with head and neck cancer (HNC) (Dukes et al., 2020). HNC are increasingly becoming an issue for global health in low-income countries, where diagnostic standard practices are not available. To this end, a SERS sensing method based on magnetic capture was developed to diagnose squamous cell carcinoma (SCC) by detecting cytokines 14 RNA (a known biomarker). The method uses nanorattles and magnetic nanoparticles, as discussed above. The described sensing mechanism is shown in Figure 2I. The detection was performed on RNA extracted directly from tissue samples collected during surgery and the results showed a high diagnostic accuracy for SCC diagnosis, without the need for any target amplification. The assay was read with a portable Raman system of the size of a small smartphone, showing that this method can be deployed for POCT and potentially be used in global health settings.

### Advances in Homogeneous Surface-Enhanced Raman Scattering Sensing Assays

Homogenous sensing is a specific SERS sensing mechanism that works autonomously. This definition means that the sensor response is generated by the nanosensor itself rather than by aggregation or by its capture. In this mechanism, the biotarget produces a change in the conformation of a Raman reporter on the surface of the SERS nanoparticle changing the observed SERS signal. Since everything happen on the surface of the nanoparticle, there is no need for washing steps to observe an accurate sensor response. The characteristic of this sensing strategy have been used to move towards *in vivo* SERS sensing because of the autonomous nature of the mechanism (Wang et al., 2018; Crawford et al., 2019). However, such characteristic combined to the simplicity of the mechanism (single step) makes it ideal for applications outside of lab settings, where multiple assay steps are not easily preformed.

One example of homogenous SERS sensors is the “molecular sentinels” (MS) that can detect nucleic acid biotargets (e.g., DNA, mRNA, and miRNA) (Wang and Vo-Dinh, 2009; Wang et al., 2015; Wang et al., 2016). Molecular sentinels work by having functional DNA on the surface of a nanoparticle. In the presence of the target, the functional DNA changes conformation. The conformation change is observed by having a Raman reporter placed at the end of the DNA strand. The distance of the reporter to the particle is varied during the mechanism, producing a change in the SERS signal. This mechanism is depicted in Figure 2II. Recently this type of sensors has been used to detect specific sequences in RNA extracted from tissue samples, extracted from plants and directly in cells (Strobbia et al., 2019; Crawford et al., 2020; Dardir et al., 2020).

Recently, the sensors were used with functional DNA reactive to the presence of miRNA-21 (Crawford et al., 2020). Such sensors were incubated with RNA extracted from tissue samples. The sensor response was compared to PCR analysis of miRNA-21 and showed low signal for healthy tissue and increasing signal for Barrett’s esophagus (precancer) and tumor tissues. This work demonstrated how homogenous sensors can be used to detect miRNA biomarkers in clinical samples. Because this assay only needs mixing of RNA samples with the homogenous sensors, it can be a valuable POCT methods due to its simplicity.

A similar approach was used in samples extracted from plants (Strobbia et al., 2019). In this application, homogeneous sensors were functionalized at the end of an optical fiber and used by dipping the fiber in the sample to analyze. This strategy was used to detect the overexpression of miRNA-156, a miRNA important in biomass production. This fiber-homogenous sensor combination can become a powerful tool to detect plant genomics and potential infections at the point of need. The addition of the fiber permits to increase the robustness and usability of this sensing method. Note that in both cases the sensors had RNA extracts as samples and further development should focus on strategy to apply the sensors to untreated or minimally treated samples.

This type of homogenous sensing mechanism has also been adapted to detect biomolecules instead of RNA by taking advantage of aptamer binding sequences. Aptamer sequences are DNA or RNA sequences selected for their affinity to a specific molecule through a process known as SELEX (Tuerk and Gold, 1990). Recent works demonstrated the detection of an endocrine disrupting chemical (i.e., BPA) directly in water samples (Chung et al., 2015). This is an important development as it expand these powerful sensing mechanism beyond biomedical applications into environmental analysis, where POCT is critical.

## Conclusion and Perspectives

In summary, each sensing mechanism has unique features that make them useful for specific POCT applications. Aggregation-based sensors have the strongest SERS signal response, which give them the highest sensitivity among these sensing mechanisms. False-positive signal due to unspecific aggregation can limit the limit-of-detection of this this type of sensors. Paper-based and magnetic methods are advantageous as they can be integrated in automated fluidic devices. The sensitivity of these mechanisms is limited. Moreover, the increased complexity given by fluidic devices should be gauged against the simplicity of non-automated solution-based sensing mechanism (aggregation-based and homogenous), especially considering POCT applied in resource-limited settings. While not as sensitive as aggregation-based methods, homogenous sensors are very reliable and have shown significant pre-clinical results. Furthermore, the homogenous mechanism makes them ideal to be used *in vivo*, *in situ* or as single-pot sensors, avoiding complexity of multiple steps and sample preparation, ideal for POCT.

In conclusion, we discussed several recent advances that are working towards the translation of SERS sensing to the POC. POCT can benefit from the use of SERS sensing that can expand the current procedures by offering methods with multiplexed and accurate detection of biomarkers. The specific application will dictate the most appropriate sensing mechanism to use based on cost, settings and sample. Several of the works described herein were applied to clinical samples. The next step into this translation is the validation of these methods and the full integration into devices. Future works should focus on detecting biomarkers directly on clinical samples at the POC and working in global health settings, where these sensors can have the biggest impact. Finally, POCT can also be critical in changing the current practice for environmental analysis, which requires sending samples to a centralize lab. Sensors can decentralize environmental analysis offering increased surveillance of harmful contaminants and SERS sensing could play an important role in this paradigm shift.
